# Supplementary material for: Deep learning predictions of TCR-epitope interactions reveal epitope-specific chains in dual alpha T cells
Source: Nat Commun. 2024 Apr 13;15:3211. doi: 10.1038/s41467-024-47461-8 (PMC11016097; doi:10.1038/s41467-024-47461-8)
Supplement: Supplementary file 3 — Reporting Summary [file 41467_2024_47461_MOESM3_ESM.pdf]

nature portfolio

Corresponding author(s): David Gfeller

Last updated by author(s): Mar 31, 2024

Reporting Summary

Nature Portfolio wishes to improve the reproducibility of the work that we publish. This form provides structure for consistency and transparency in reporting. For further information on Nature Portfolio policies, see our Editorial Policies and the Editorial Policy Checklist.

Statistics

For all statistical analyses, confirm that the following items are present in the figure legend, table legend, main text, or Methods section.

n/a	Confirmed	
☐	☒	The exact sample size (n) for each experimental group/condition, given as a discrete number and unit of measurement
☐	☒	A statement on whether measurements were taken from distinct samples or whether the same sample was measured repeatedly
☐	☒	The statistical test(s) used AND whether they are one- or two-sided<br>*Only common tests should be described solely by name; describe more complex techniques in the Methods section.*
☒	☐	A description of all covariates tested
☒	☐	A description of any assumptions or corrections, such as tests of normality and adjustment for multiple comparisons
☐	☒	A full description of the statistical parameters including central tendency (e.g. means) or other basic estimates (e.g. regression coefficient) AND variation (e.g. standard deviation) or associated estimates of uncertainty (e.g. confidence intervals)
☐	☒	For null hypothesis testing, the test statistic (e.g. F, t, r) with confidence intervals, effect sizes, degrees of freedom and P value noted<br>*Give P values as exact values whenever suitable.*
☒	☐	For Bayesian analysis, information on the choice of priors and Markov chain Monte Carlo settings
☒	☐	For hierarchical and complex designs, identification of the appropriate level for tests and full reporting of outcomes
☐	☒	Estimates of effect sizes (e.g. Cohen's d, Pearson's r), indicating how they were calculated

Our web collection on statistics for biologists contains articles on many of the points above.

Software and code

Policy information about availability of computer code

Data collection	No specific software was used to download the TCR-epitope sequence data from publicly available databases. FACS samples were acquired with IntelliCyt iQue® Screener PLUS (Bucher BiotecSartorius) flow cytometer.
Data analysis	The data analysis was done in python(v.3.9.7) using dedicated libraries such as biopython v.1.79, pandas v.1.5.2. . Several datasets used in this study were generated with the Cell Ranger Single Cell Software Suite by 10X Genomics. We further processed those data with dedicated python libraries such as Scanpy v.1.9.3, Scripy v.0.10.0 and scrublet v.0.2.3. FACS data were analyzed with FlowJo 10.8.1 (TreeStar).

For manuscripts utilizing custom algorithms or software that are central to the research but not yet described in published literature, software must be made available to editors and reviewers. We strongly encourage code deposition in a community repository (e.g. GitHub). See the Nature Portfolio guidelines for submitting code & software for further information.

Data

Policy information about availability of data

All manuscripts must include a data availability statement. This statement should provide the following information, where applicable:

- Accession codes, unique identifiers, or web links for publicly available datasets
- A description of any restrictions on data availability
- For clinical datasets or third party data, please ensure that the statement adheres to our policy

TCR-pMHC pairs were collected from the publicly available datasets VDJdb, (https://vdjdb.cdr3.net/), IEDB (https://www.iedb.org/), McPAS (http://friedmanlab.weizmann.ac.il/McPAS-TCR/), and the 10X Genomics dataset (https://pages.10xgenomics.com/rs/446-PBO-704/

images/10x_AN047_IP_A_New_Way_of_Exploring_Immunity_Digital.pdf). Additional data for Mus musculus were retrieved from two recent studies (https://www.ncbi.nlm.nih.gov/geo/query/acc.cgi?acc=GSE182320 and https://www.ncbi.nlm.nih.gov/geo/query/acc.cgi?acc=GSE201730). A total of 17,715 different TCRs for 146 pMHCs were collected. This dataset together with the MixTCRpred code are available at https://github.com/GfellerLab/MixTCRpred and on Zenodo 10.5281/zenodo.10806391. The pre-trained MixTCRpred models for the 146 pMHCs reported in the manuscript are available at https://zenodo.org/record/7930623.

Research involving human participants, their data, or biological material

Policy information about studies with human participants or human data. See also policy information about sex, gender (identity/presentation), and sexual orientation and race, ethnicity and racism.

Reporting on sex and gender	N/A
Reporting on race, ethnicity, or other socially relevant groupings	N/A
Population characteristics	N/A
Recruitment	N/A
Ethics oversight	N/A

Note that full information on the approval of the study protocol must also be provided in the manuscript.

Field-specific reporting

Please select the one below that is the best fit for your research. If you are not sure, read the appropriate sections before making your selection.

[x] Life sciences [ ] Behavioural & social sciences [ ] Ecological, evolutionary & environmental sciences

For a reference copy of the document with all sections, see nature.com/documents/nr-reporting-summary-flat.pdf

Life sciences study design

All studies must disclose on these points even when the disclosure is negative.

Sample size	Experiments were performed to access the in-vitro binding of specific TCRs towards specific target epitopes. Therefore no sample size calculation was performed
Data exclusions	No data were excluded from the analysis
Replication	No replication was performed for the in-vitro binding assays
Randomization	We tested the the in-vitro binding of specific TCRs towards specific target epitopes. No randomization was performed
Blinding	We tested the the in-vitro binding of specific TCRs towards specific target epitopes. The blinding concept is not applicable

Reporting for specific materials, systems and methods

We require information from authors about some types of materials, experimental systems and methods used in many studies. Here, indicate whether each material, system or method listed is relevant to your study. If you are not sure if a list item applies to your research, read the appropriate section before selecting a response.

Materials & experimental systems

n/a	Involved in the study	
[ ]	[x]	Antibodies
[ ]	[x]	Eukaryotic cell lines
[x]	[ ]	Palaeontology and archaeology
[x]	[ ]	Animals and other organisms
[x]	[ ]	Clinical data
[x]	[ ]	Dual use research of concern
[x]	[ ]	Plants

Methods

n/a	Involved in the study	
[x]	[ ]	ChIP-seq
[ ]	[x]	Flow cytometry
[x]	[ ]	MRI-based neuroimaging

Antibodies

Antibodies used	Antibodies were titrated for optimal staining. For TCR screening we used the following fluorophore conjugated antibodies/multimers: anti-hCD3 APC Fire 50 (SK7, Biolegend Cat# 641415, 0.4uL in 50uL); anti-hCD8 FITC (SK-1 Biolegend, Cat# 344704, 0.15uL in 50uL); anti-hCD4 PE-CF594 (RPA-T4, BD Bioscience Cat# 562281, 0.4uL in 50uL); anti-mouse TCRβ-constant APC (H57-597, Thermo Fisher Scientific, Cat# 17-5961-81, 0.6uL in 50uL); pMHC-multimer-PE (HLA-A*02:01,GILGFVFT and HLA-A*02:01,ELAGIGILTV in-house synthezised, 1uL in 50uL); viability dye Aqua (L34966, Thermo Fisher Scientific, 0.15uL in 50uL staining mix in PBS).
Validation	Antibodies' concentrations validation was empirically determined in the lab, as per manufacturers' instructions. All primary antibodies were validated and titrated with human PBMCs or additional irrelevant cells using IntelliCyt iQue flow cytometer.

Eukaryotic cell lines

Policy information about cell lines and Sex and Gender in Research

Cell line source(s)	Jurkat T cells were provided by Promega (J1601, T Cell Activation Bioassay (NFAT))
Authentication	Cells were not authenticated after the purchase.
Mycoplasma contamination	Cells were mycoplasma negative as assessed by PCR.
Commonly misidentified lines (See ICLAC register)	No commonly misidentified lines were used in the study

Plants

Seed stocks	N/A
Novel plant genotypes	N/A
Authentication	N/A

Flow Cytometry

Plots

Confirm that:

- [x] The axis labels state the marker and fluorochrome used (e.g. CD4-FITC).
- [x] The axis scales are clearly visible. Include numbers along axes only for bottom left plot of group (a 'group' is an analysis of identical markers).
- [x] All plots are contour plots with outliers or pseudocolor plots.
- [x] A numerical value for number of cells or percentage (with statistics) is provided.

Methodology

Sample preparation	200000 TCR-transfected Jurkat cells were washed once in PBS, resuspended in 50uL of FACS buffer (5mM EDTA, 0.2% azide, 0.2% BSA in PBS) containing 1uL of multimer and incubated for 30 min at RT. Cells were washed once in PBS and resuspended in 50uL of PBS containing LIVE/DEAD dye and the antibody cocktail for cell surface staining (Antibodies section of the present document). Cells were incubated at 4°C for 30 minutes and washed twice before acquisition.
Instrument	IntelliCyt iQue® Screener PLUS (Bucher BiotecSartorius) flow cytometer
Software	FlowJo 10.8.1
Cell population abundance	All cells were acquired for the analysis

Gating strategy

Homogeneous lymphatic cells have been gated on the FSC-SSC dot-plot. Doublets have been excluded by gating on a FSC-H/FSC-A dot-plot. All viable cells were identified on a aqua/multimer dot-plot, followed by CD3 selection (dot-plot: CD3/multimer). CD4- were selected (dot-plot CD4/multimer). All CD8 cells were gated on a CD8/multimer dot-plot. Finally multimer + cells were shown on CD8/multimer dot-plots (figures).

☒ Tick this box to confirm that a figure exemplifying the gating strategy is provided in the Supplementary Information.
